# Effects on hemodynamics and gas exchange of omega-3 fatty acid-enriched lipid emulsion in acute respiratory distress syndrome (ARDS): a prospective, randomized, double-blind, parallel group study

**DOI:** 10.1186/1476-511X-7-39

**Published:** 2008-10-23

**Authors:** Joan Sabater, Joan Ramon Masclans, Judit Sacanell, Pilar Chacon, Pilar Sabin, Merce Planas

**Affiliations:** 1 Intensive Medicine Department, Hospital General Universitari Vall d'Hebron, Barcelona, Spain; 2 Biochemistry Department, Hospital General Universitari Vall d'Hebron, Barcelona, Spain; 3 Pharmacy Department, Hospital General Universitari Vall d'Hebron, Barcelona, Spain; 4 Nutritional Support Unit, Hospital General Universitari Vall d'Hebron, Barcelona, Spain; 5 Departament de Medicina, Universitat Autonoma de Barcelona, Spain

## Abstract

**Introduction:**

We investigated the effects on hemodynamics and gas exchange of a lipid emulsion enriched with omega-3 fatty acids in patients with ARDS.

**Methods:**

The design was a prospective, randomized, double-blind, parallel group study in our Intensive Medicine Department of Vall d'Hebron University Hospital (Barcelona-Spain). We studied 16 consecutive patients with ARDS and intolerance to enteral nutrition (14 men and 2 women; mean age: 58 ± 13 years; APACHE II score: 17.8 ± 2.3; Lung Injury Score: 3.1 ± 0.5; baseline PaO_2_/FiO_2 _ratio: 149 ± 40). Patients were randomized into 2 groups: Group A (n = 8) received the study emulsion Lipoplus^® ^20%, B.Braun Medical (50% MCT, 40% LCT, 10% ω-3); Group B (n = 8) received the control emulsion Intralipid^® ^Fresenius Kabi (100% LCT). Lipid emulsions were administered during 12 h at a dose of 0.12 g/kg/h. Measurements of the main hemodynamic and gas exchange parameters were made at baseline (immediately before administration of the lipid emulsions), every hour during the lipid infusion, at the end of administration, and six hours after the end of administration lipid infusion.

**Results:**

No statistically significant changes were observed in the different hemodynamic values analyzed. Likewise, the gas exchange parameters did not show statistically significant differences during the study. No adverse effect attributable to the lipid emulsions was seen in the patients analyzed.

**Conclusion:**

The lipid emulsion enriched with omega-3 fatty acids was safe and well tolerated in short-term administration to patients with ARDS. It did not cause any significant changes in hemodynamic and gas exchange parameters.

**Trial registration:**

ISRCTN63673813

## Introduction

The lipid emulsions generally used in the parenteral nutrition of critically ill patients are rich in long-chain triglycerides (LCT), especially linoleic acid (polyunsaturated series 6 fatty acid; PUFA n-6, 18:2 n-6). These preparations guarantee an optimal energy supply and prevent deficiency in essential fatty acids[1]. These fatty acids, aside from producing adverse metabolic effects (transitory hyperlipidemias), can alter pulmonary gas exchange due to their potentially proinflammatory properties [2].

The MCT/LCT emulsions are oxidized faster and provide fewer amounts of PUFAs than a LCT emulsions. While MCT emulsions do not interfere with eicosanoids synthesis, it has been considered as neutral. Thus MCT/LCT emulsions have been associated with a lower risk of lipid peroxidation and fewer alterations of membrane structures[3].

Polyunsaturated fatty acids of the n-3 series (PUFA n-3) are precursors of biologically active substances, e.g. the series 3 and 5 eicosanoids. These molecules use the same metabolic routes and compete for the same elongases and desaturases as linoleic and arachidonic, but ultimately they are mediators that have a much less active biological profile than linoleic acid derivatives[4]. In 146 patients with ARDS treated with an enteral diet rich in eicosapentaenoic acid, γ-linolenic, and antioxidants, an improved PaO_2_/FiO_2 _ratio, and a reduction in pulmonary inflammatory response, frequency of new organ failures, days of mechanical ventilation and long of stay in intensive care were observed[5]. In 100 patients with acute pulmonary injury receiving a similar enteral diet, oxygenation was better, pulmonary compliance improved, and there was less need for mechanical ventilation[6]. The administration of LCT emulsions has been associated with changes in pulmonary function that depend on pulmonary disease, dose, duration, and speed of administration, and kind of infused lipids [7,8].

Many patients with ARDS require sedoanalgesia and myorelaxation, inducing intestinal ileus and making intolerance to enteral nutrition. We hypothesized that adding n-3 to a MCT/LCT emulsion used in parenteral nutrition, should be beneficial because of the reduced amount of PUFA, would be less toxic than a lipid emulsion based on soybean oil, and that the addition of fish oil would be protective because of its anti-inflammatory properties[4]. The present study evaluated the hemodynamic changes and variations in pulmonary gas exchange that occurred in patients with ARDS given an intravenous lipid emulsion enriched with n-3 fatty acids.

## Materials and methods

### Patients

We studied 16 consecutive patients with ARDS in the first 48 hours of admission and intolerance of enteral nutrition defined by gastric aspirates higher than 300 ml. Patients were enrolled after approval of the study by the Committee of Clinical Trials of the Vall d'Hebron General University Hospital of Barcelona. All the patients or their representatives signed an informed consent before enrollment in the study. The inclusion criteria were: bilateral pulmonary infiltrates of sudden onset in the chest radiograph, PaO_2_/FiO_2 _less than 200, and pulmonary capillary pressure less than 18 mm Hg[9]. Table 1 shows the clinical characteristics of the patients studied. Patients were excluded for age younger than 18 or older than 85 years, pregnancy, liver failure, HIV positivity, leukopenia (< 3500 mm^3^), thrombocytopenia (< 100,000 mm^3^), severe renal insufficiency (creatinine > 6 mg/dl) or need for renal dialysis, signs of heart failure, transplantation, multiple blood transfusions, participation in other clinical trials simultaneously or in the last 60 days, treatment with nitrous oxide or corticoids (prednisolone 2 mg/kg/d or equivalent), multiple organ failure, severe dyslipidemia, or propofol treatment.

**Table 1:** Clinical characteristics of patients

**Diagnosis**	**Sex**	**Age**	**PaO_2_/FiO_2_**	**LIS**	**APACHEII**	**PEEP**	**Outcome**
**Group A: n-3**							

1. Pneumonia	M	74	98	4	18	7	D

2. Pneumonia	M	48	166	2.3	17	8	D

3. Pneumonia	M	64	133	3.6	18	12	D

4. Sepsis	M	50	189	3	15	10	L

5. Smoke aspiration	M	40	132	3	16	11	L

6. Bronchoaspiration	M	72	84	2.6	21	8	D

7. Mediastinitis	M	70	147	3.3	19	10	L

8. Bronchoaspiration	M	36	183	2.5	15	7	L

**Total Group A:**	**8 M**	**56 ± 15**	**141 ± 37**	**3 ± 0.5**	**17.4 ± 2.1**	**9.1 ± 1.9**	**4D/4L**

**Group B: LCT**							

1. Pancreatitis	F	52	152	3	16	11	L

2. Bronchoaspiration	M	57	198	4	21	15	L

3. Bronchoaspiration	M	56	177	2.6	17	9	D

4. Status postpneumectomy	M	63	60	4	19	10	L

5. Pneumonia	M	71	181	3	18	10	L

6. Pneumonia	M	43	186	3.3	14	8	L

7. Pneumonia	M	53	153	3	19	10	D

8. Pneumonia	F	81	157	3	22	9	L

**Total Group B**	**6M/2F**	**59 ± 12**	**158 ± 43**	**3.2 ± 0.5**	**18.2 ± 2.6**	**10.3 ± 2.1**	**2D/6L**

**Total n:16**	**14M/2F**	**58 ± 13**	**149 ± 40**	**3.1 ± 0.5**	**17.8 ± 2.3**	**9.7 ± 2**	**6D/10L**

LIS: Lung Injury Score. APACHEII: Acute Physiology and Chronic Health Evaluation. PEEP: Positive End Expiratory Pressure. M: male. F: female. D: dead. L: alive.

Patients were sedated with morphine and midazolam and muscular relaxation was used as needed. All patients were ventilated mechanically (Mallinckrodt Puritan Bennett 7200 series, Carlsbad, CA, USA). Monitorization included continuous electrocardiography (ECG), heart rate (HR), pulse oximetry (SpO_2_), invasive blood pressure, pulmonary artery catheterization, and continuous cardiac output by thermodilution.

### Measurements

A central venous catheter was inserted to administer the lipid emulsions. The basic parameters of pulmonary mechanics were monitored with the respirator and external monitoring. Cardiac output (CO) was measured by thermodilution. Systemic blood pressure (SBP), central venous pressure (CVP), pulmonary artery pressure (PAP), and pulmonary capillary pressure (PCP) were monitored by transducers placed on the middle anteroposterior chest, zeroed to atmospheric pressure, and calibrated electronically. Systemic (SVR) and pulmonary (PVR) vascular resistance, O_2 _transport (DO_2_), and pulmonary Q_s_/Q_t _were calculated with the usual formulas. Blood samples were drawn under anaerobic conditions through catheters placed in the radial artery and pulmonary artery. Oxygen (PaO_2_) and carbon dioxide (PaCO_2_) partial pressure, pH, and the concentration and mixed arterial and venous saturation of haemoglobin were measured using standard electrodes.

### Study design

In the first 48 hours after the diagnosis of ARDS and before receiving artificial nutrition, patients were randomized into two different groups: Group A (n = 8) received the study emulsion Lipoplus^® ^20%, B.Braun Medical (50% MCT, 40% LCT, 10% ω-3); Group B (n = 8) received the control emulsion Intralipid^® ^20% Fresenius Kabi (100% LCT). The lipid emulsions were administered during 12 hours at a rate of 0.12 g/kg/h. During the lipid perfusion no recruitment maneuvres were done.

Measurements were made at baseline (immediately before the administration of lipid emulsions, every hour during the lipid infusion, at the end of lipid perfusion and six hours latter. Data reported in tables: baseline (t 0), six hours of perfusion (t 6); end of perfusion (t 12), and six hours after perfusion (t 18). Basic parameters of pulmonary mechanics, arterial and mixed venous gas analysis, hemodynamic parameters, and oxygen transport were measured at all stages.

### Statistical analysis

The data were imported from Access databases to SPSS. The following statistical parameters were calculated for numerical variables: mean and standard desviation. Differences in the means were analyzed by the T-test. Differences in time of the different parameters studied, depending on the type of emulsion, were assessed by repeated-measure analysis of variance (ANOVA). The level of statistical significance was defined as p < 0.05.

## Results

### Characteristics of the study population

At baseline, the groups were comparable (table 1, 2 and 3). Probably because of the exclusion criteria, the majority of the patients studied are patients with primary pulmonary disease as primary etiology of the ARDS. Although mortality was 50% in group A and 25% in group B, no significant differences in survival between the two groups were found. Probably the differences in mortality between groups are a simple size question. The global mortality rate in the ICU during the study period time was 22%; in this ICU no patients with coronary disease or cardiovascular surgery were admitted. All patients were sedated with midazolam and morphine in continuous perfusion and underwent controlled-volume ventilation. No significant modifications in the ventilation parameters were necessary during the study period time. There were no significant changes between the two groups.

**Table 2:** Hemodynamic parameters

	**T-0 h (baseline, preinfusion)**	**T-6 h**	**T-12 h (final infusion)**	**T-18 h (6 h postinfusion)**
**HR**				

A: n-3	91 ± 19	101 ± 20	100 ± 21	91 ± 25

B: LCT	95 ± 13	93 ± 12	95 ± 12	84 ± 16

**CO**				

A: n-3	7.8 ± 2.8	8.4 ± 2.7	8.5 ± 2.7	7.8 ± 3

B: LCT	6.9 ± 1.3	7.3 ± 1.9	6.8 ± 1.6	6.1 ± 1.3

**SAP**				

A: n-3	127 ± 19	113 ± 14	117 ± 15	131 ± 24

B: LCT	126 ± 9	126 ± 10	124 ± 14	121 ± 14

**MPAP**				

A: n-3	26 ± 5	29 ± 4	28 ± 7	28 ± 6

B: LCT	30 ± 7	31 ± 7	31 ± 6	28 ± 7

**PW**				

A: n-3	13 ± 4*	13 ± 2	14 ± 3	14 ± 4

B: LCT	16 ± 2	17 ± 2	17 ± 2	16 ± 2

**PVR**				

A: n-3	162 ± 122	187 ± 98	155 ± 68	162 ± 92

B: LCT	147 ± 84	142 ± 102	138 ± 79	134 ± 77

**SVR**				

A: n-3	822 ± 328	644 ± 194	629 ± 228	864 ± 376

B: LCT	863 ± 217	840 ± 274	884 ± 298	932 ± 238

**DO_2_**				

A: n-3	1074 ± 340	1171 ± 406	1174 ± 372	1066 ± 518

B: LCT	966 ± 224	1023 ± 330	936 ± 227	856 ± 225

**VO_2_/DO_2_**				

A: n-3	0.22 ± 0.04	0.24 ± 0.05	0.23 ± 0.04	0.25 ± 0.06

B: LCT	0.26 ± 0.05	0.25 ± 0.04	0.27 ± 0.03	0.25 ± 0.04

**Q_s_/Q_t_**				

A: n-3	33 ± 10	31 ± 8	33 ± 11	28 ± 7

B: LCT	28 ± 11	27 ± 6	25 ± 8	25 ± 9

**BT**				

A: n-3	38.1 ± 1	38 ± 0.9	38.1 ± 0.8	37.6 ± 1.2

B: LCT	37.6 ± 0.4	37.5 ± 0.6	37.6 ± 0.4	37.1 ± 1

HR: heart rate, CO: cardiac output, SAP: systolic artery pressure, MPAP: mean pulmonary artery pressure, PW: pulmonary artery occlusion pressure, PVR: pulmonary vascular resistances, SVR: systemic vascular resistances DO_2_: oxygen delivery, Q_s_/Q_t_: venous admixture ratio, BT: body temperature.

* p < 0.05 between baseline groups

**Table 3:** Respiratory parameters

	**T-0 h (baseline, preinfusion)**	**T-6 h**	**T-12 h (final infusion)**	**T-18 (6 h postinfusion)**
**PaO_2_/FiO_2_**				

A: n-3	137 ± 35	143 ± 44	161 ± 87	162 ± 59

B: LCT	158 ± 43	158 ± 47	184 ± 84	192 ± 66

**pH**				

A: n-3	7.42 ± 0.07	7.41 ± 0.07	7.38 ± 0.06	7.41 ± 0.07

B: LCT	7.40 ± 0.07	7.41 ± 0.06	7.40 ± 0.06	7.41 ± 0.1

**PaO_2_**				

A: n-3	90 ± 25	99 ± 17	127 ± 85	106 ± 30

B: LCT	101 ± 32	99 ± 31	113 ± 59	122 ± 42

**PaCO_2_**				

A: n-3	42 ± 2.3	41.2 ± 4.9	44.6 ± 4.7	43.8 ± 5

B: LCT	40.5 ± 7.6	41.9 ± 8	41.3 ± 5	40.5 ± 6

**ST BIC**				

A: n-3	26.6 ± 4.5	25.5 ± 4.7	25.6 ± 4.1	26.6 ± 3.4

B: LCT	24.5 ± 4	25.9 ± 5.4	25 ± 3.1	25.5 ± 5.2

**BE**				

A: n-3	2.4 ± 4.6	0.9 ± 4.8	0.6 ± 4.5	1.9 ± 4.4

B: LCT	0.1 ± 4.4	1.2 ± 5	0.3 ± 4	0.9 ± 5.5

BE: base excess, ST BIC: standard bicarbonate.

### Effect on gas exchange

After the administration of lipid emulsions, no significant modifications, intragroup or intergroup, in gas exchange or respiratory variables were observed (table 3)

### Effect on hemodynamics

No significant differences were observed in either blood pressure or any other parameter studied during the administration of the lipid emulsions. Comparison of the changes between the two groups only yielded significant findings for pulmonary capillary pressure, which was lower at the end of emulsion infusion in group A, which received the lipid emulsion enriched with n-3 fatty acids. The fluid balance and medications used were similar in both groups (table 2).

## Discussion

The results of the present study suggest that none of the lipid emulsions administered at the rate and for the duration studied here produced gas exchange or hemodynamic disturbances in patients with ARDS. This suggests that the emulsions were clinically safe under these conditions. The changes in gas exchange induced by lipid emulsions in patients with ARDS depend on various factors such as the rate of administration and duration, composition of the lipid emulsions (as precursors of prostanoid synthesis), and the characteristics of the study populations (tables 4a, and 4b).

**Table 4:** Cardiopulmonary effects of LCT and MCT/LCT in humans


**A (LCT)**				

**Author**	**Emulsion**	**Infusion rate**	**Patients**	**Results**

Venus[11]	20% LCT	100 g/10 h	Sepsis Non-sepsis	↑ MPAP, ↑ Q_s_/Q_t_

Venus[12]	20% LCT	100 g/8 h	ARDS	↓ PaO_2_/FiO_2_ ↑ MPAP, ↑ Q_s_/Q_t_, ↑ PVR

Hwang[8]	10% LCT	50 g/4 h	Healthy ARDS Pneumonia COPD	↓ Q_s_/Q_t_, ↑ PaO_2_/FiO_2_ ↑ Q_s_/Q_t_, ↓ PaO_2_/FiO_2_ No changes No changes

Mathru[7]	20% LCT	100 g/10 h 100 g/5 h	ARDS ARDS	↑ Q_s_/Q_t_, =MPAP =Q_s_/Q_t_, ↑ MPAP

				
**B (MCT/LCT)**				
				

Radermacher[13]	20% MCT/LCT	50 g/4 h	Sepsis	No changes

Fiaccadori[14]	20% MCT/LCT	3.3 mg/kg min	Heart surgery	No changes

Masclans[17]	20% LCT 20% MCT/LCT	2 mg/kg/min 12 h 2 mg/kg/min 12 h	ARDS ARDS	↑ CO No changes

Smyrniotis[18]	20% LCT 20% MCT/LCT	50% non-prot cal	Pancreatitis with ARDS	↑ MPAP, ↑ Q_s_/Q_t_, and ↓ PaO_2_/FiO_2_ ↑ VO_2_, ↑ CO

Chassard[19]	20% LCT 20% MCT/LCT	3 mg/kg/min 8 h 3 mg/Kg/min 8 h	Sepsis	No changes ↑ VO_2_

Faucher[20]	20% LCT 20% MCT/LCT	1 ml/kg/h 6 h 1 ml/kg/h 6 h	ARDS	↑ PaO_2_/FiO_2_, ↑ DO_2_

Lekka[16]	20% MCT/LCT Placebo		ARDS: lipids Placebo	↓ PaO_2_/FiO_2_, ↓ Comp, ↓ PVR

VO_2_: oxygen consumption, Comp: compliance.

Analysis of the cardiopulmonary effects of lipid emulsions in our previous studies and the literature yields contradictory findings. In relation to the administration of LCT enriched emulsions; in healthy adults the administration of 4 hours infusion of Intralipid was followed by a significant increase in systolic and diastolic blood pressure as well as heart rate[10]. 500 ml of LCT 20% infusion increases pulmonary artery pressure and venous admixture in critically ill patients. The changes observed were temporary and coincidental with serum lipemia[11]. The same authors, in ARDS patients observed that an infusion of 20% LCT at 3.0 ± 0.3 mg/kg/min was followed by a transitory reduction in PaO_2_/FIO_2_, and an increase in MPAP, pulmonary vascular resistance and pulmonary venous admixture[12]. In different type of patients, the administration of 500 ml of LCT 10% in 4 h resulted in a reduction of the PaO_2_/FiO_2 _ratio and increased P(A-a)O_2 _and intrapulmonary shunting only in patients with ARDS[8]. Slow infusion of an LCT 20% emulsion (500 ml in 10 h) to patients with pulmonary injury increased intrapulmonary shunting, possibly by increasing the production of endogenous vasodilators, while rapid administration of the emulsion (500 ml in 5 h) increased MPAP possibly due to synthesis of endogenous vasoconstrictors[7].

Athough, theorically, the administration of emulsions enriched with MCT/LCT should have a neutral effect due to the few amount of PUFAs, the review of the literature was not concordant. In septic patients MCT/LCT administration did not induce any alterations in pulmonary hemodynamics, gas exchange, or distribution of ventilation/perfusion[13]. No hemodynamic neither respiratory modifications were observed in patients immediately after valvular surgery[14] or in critically ill patients with mechanical ventilation[15]. In contrast, deterioration of oxygenation, compliance, and pulmonary vascular resistance was observed after administering MCT/LCT to patients with ARDS[16]. Our group analyzed the gas exchange and pulmonary haemodynamic responses to LCT or MCT/LCT in ARDS patients. LCT infusion was followed by an increment in oxygen consumption without alterations in arterial oxygenation probably by the benefficial effect of the increase in cardiac output observed[17]. Also in patients with ARDS, while LCT infusion was followed by an increase in MPAP, and in pulmonary venous admixture, and a decrease in arterial PaO_2_, MCT/LCT infusion produced an increase in oxygen consumption, cardiac output and CO_2 _production[18]. In critically ill patients with mechanical ventilation, the infusion of MCT/LCT vs LCT was accompanied by increased oxygen consumption and minute ventilation[19]. A benefit in oxygenation and increased cardiac output was demonstrated in patients with ARDS treated with MCT/LCT lipid emulsions compared to LCT[20]. From the revision of different data available using LCT, MCT/LCT fat emulsions we observed contradictory effects on pulmonary and hemodynamic variables; probably due to the different pathologic situations, and the amount and rate of lipid emulsions administered.

It seems that soy bean lipid emulsions represent an imbalanced fatty acid supply, with an overabundance of n-6 PUFAs. Emulsions that include MCT and fish oil as a partial replacement for soy bean are now available, and evidence to data is that these emulsions offer some advantages over the use of soy bean alone. At the neutral metabolic effects of MCTs they added the beneficial properties of n-3 fatty acids, with diminished circulating concentrations of inflammatory eicosanoids [21-23]. Several studies in surgical and septic patients promotes the use of omega-3 lipids in these clinical situations, however few data from randomized controlled trials are available [24,25]. In patients with ARDS, our group showed that intravenous administration of an n-3 enriched lipid emulsion was accompanied by the synthesis of a smaller amount of proinflammatory mediators compared to LCT 100% emulsion[26].

## Conclusion

In our series of patients with ARDS, no significant changes were observed in hemodynamics or gas exchange, which demonstrated the safety of n-3 enriched lipid emulsions in these critical patients. The absence of theorically expected changes related to modifications observed in the eicosanoid profile may be due to the short duration of treatment with lipid infusion. However, now that it has been demonstrated that the n-3 enriched emulsion is safe, it can be used for studies in patients with ARDS who require PN. More prolonged use may reveal improvements in the clinical evolution of this group of patients, who traditionally bear a heavy morbidity and mortality.

## Key messages

• Lipid n-3 enriched emulsions are safe in short term treatments with ARDS patients.

• Despite theorical benefits in eicosanoid profile, the absence of clinical benefit could be due to the short treatment duration. Further studies are needed with longer infusion treatments.

## List of abbreviations

ARDS: Acute respiratory distress syndrome; MCT: Medium-chain triglycerides; LCT: Long-chain triglycerides; ω-3: Omega 3; PUFA n-6: Polyunsaturated series 6 fatty acid; n-3 fatty acids: Fatty acids of the n-3 series; PUFA n-3: Polyunsaturated fatty acids of the n-3 series; EPA 20:5 n-3: Eicosapentaenoic acid; DHA 22:5 n-3: Docosahexaenoic acid ECG: Electrocardiography; HR: Heart rate; SpO_2_: Pulse oximetry; CO: Cardiac output; SBP: Systemic blood pressure; CVP: Central venous pressure; PAP: Pulmonary artery pressure; PCP: Pulmonary capillary pressure; SVR: Systemic vascular resistance; PVR: Pulmonary vascular resistance; DO_2_: O_2 _transport; Q_s_/Q_t_: Venous admixture ratio; PaO_2_: Oxygen partial pressure in the artery; PaCO_2_: Carbon dioxide partial pressure in the artery; MPAP: Mean pulmonary artery pressure; P(A-a)O_2_: Alveolo-arterial gradient; PN: Parenteral nutrition; LIS: Lung Injury Score; APACHEII: Acute Physiology and Chronic Health Evaluation; PEEP: Positive End Expiratory Pressure.

## Competing interests

The authors declare that this study has been financed by B. Braun Medical.

## Authors' contributions

JS participated in both the study design and coordination, and drafted the manuscript. JRM participated in both the study design and coordination, and performed the statistical analysis. JS participated in both the study design and coordination. PC participated in the study design. PS participated in the study design. MP designed and coordinated the study, and reviewed the final manuscript. All authors read and approved the final manuscript.
